# Role of the skilled nursing facility in *Clostridioides difficile* infection transitions of care: A retrospective cohort study of US hospitals

**DOI:** 10.1016/j.ajic.2025.10.012

**Published:** 2025-10-24

**Authors:** Kelly R. Reveles, Kelsey A. Strey, Cory Evans, Diego Sierra, Victor Herrera, Glenn S. Tillotson

**Affiliations:** a College of Pharmacy, The University of Texas at Austin, Austin, TX; b Pharmacotherapy Education and Research Center, University of Texas Health San Antonio, San Antonio, TX; c Clinware Solutions, Inc., New Hill, NC; d GST Micro LLC, North, VA

**Keywords:** Skilled nursing facilities, Epidemiology

## Abstract

**Background:**

Skilled nursing facility (SNF) residents are at increased risk of *Clostridioides difficile* infection (CDI). The study describes the role of SNFs in CDI transitions of care among hospitalized patients in the United States.

**Methods:**

This was a retrospective cohort study using the PINC AI Healthcare Database. Older adults (65+ years) were included if they had an index, nonrecurrent CDI hospitalization between July 2019 and December 2022. Patient and CDI characteristics, health outcomes (inpatient mortality, hospital length of stay, and readmissions), and hospital costs were compared between patients admitted from or discharged to a SNF.

**Results:**

Among 86,646 index CDI hospitalizations, only 5.1% of CDI patients were admitted from a SNF; however, 28.3% were discharged to a SNF. Patients admitted from SNFs more commonly experienced inpatient mortality (13.5% vs 8.2%), all-cause readmission (31.0% vs 28.0%), higher median hospital costs ($18,610 vs $15,270), and longer median hospital length of stay (8 vs 7 days) (*P* < .05 for all). Similar trends were noted for patients discharged to a SNF.

**Conclusions:**

Older, hospitalized CDI patients originating from SNFs disproportionately experience poor health outcomes and financial burden. Over one-quarter of CDI patients were discharged to a SNF suggesting a need for higher levels of health care following CDI.

## BACKGROUND

*Clostridioides difficile* infection (CDI) is a significant public health challenge that disproportionately affects older adults. It is estimated that 70% of CDIs occur in patients older than 65 years.^1^ National CDI incidence more than doubled among hospitalized, older adults in the United States between 2001 and 2010, significantly outpacing increases in other age groups.^2^ Older adults are more likely to experience poor CDI health outcomes, including severe CDI, prolonged hospital stays, mortality, and recurrent CDI (rCDI) compared with younger adults.^2,3^ Thus, in the setting of an aging population, CDI has major implications for the demand for health care services, including hospitalizations, home care, skilled nursing, and long-term care.

Older adults are more likely to develop CDI and its complications for several reasons. Older adults experience a decline in physiological reserve (ie, frailty) and immune function with age, higher prevalence of chronic and acute comorbidities, and greater health care exposures. Poor functional and cognitive status and frailty are more common among patients who develop CDI and are predictors of severe CDI and mortality.^4–6^ A particularly high-risk population enriched for these risk factors is skilled nursing facility (SNF) residents. SNF residents are generally older and have more comorbidities and health care exposures compared with community-dwelling adults.^7,8^ In fact, SNF residents are more likely to develop CDI^9^ and 90% of SNF residents who develop CDI have underlying comorbidities. Inpatient mortality rates are significantly higher among SNF patients who develop CDI compared with non-SNF CDI patients (12.8% vs 8.2%).^4^ Antibiotics are also the most commonly prescribed medications in nursing homes; up to 70% of residents receive at least one course of systemic antibiotics per year and 40% to 75% of these antibiotics may be unnecessary or inappropriate.^10,11^

CDI may also necessitate postacute care among previously community-dwelling patients. One prior study found that patients who develop CDI are significantly more likely to be discharged to a nonhome setting compared with those who do not develop CDI.^12^ Studies have documented poor quality of life during and after CDI, with patients experiencing ongoing issues such as fatigue, anxiety, diminished ability to perform daily activities, and worsening chronic conditions.^13–15^ Furthermore, more than half of patients continue to experience post-CDI symptoms after infection resolution, highlighting the persistent and debilitating nature of the disease.^14^ The long-term impact of CDI on this population is significant and often requires intense postacute care, including rehabilitation and extended stays in long-term care facilities. With up to one-quarter of CDI patients requiring readmission,^16^ these challenges highlight the urgent need for effective care transitions to improve outcomes for this vulnerable group.

Despite the recognized importance of managing transitions of care for CDI patients originating from SNFs, a significant knowledge gap remains due to the limited studies exploring the nuances of these care transitions and their direct impact on health outcomes. The primary objective of this study was to analyze CDI transitions of care for patients admitted from and discharged to SNFs, including CDI acuity, health outcomes, and associated health care costs.

## METHODS

### Study design and setting

This was a retrospective cohort study using data from the PINC AI Healthcare Database (PHD; Premier, Inc).^17^ The PHD is an all-payer database that contains information on inpatient and outpatient visits to approximately one-quarter of all US health care systems. The PHD is nationally representative of nonprofit, nongovernmental, and community and teaching hospitals. The PHD includes information on patient, hospital, and clinical characteristics, costs and charges, laboratory data, purchase data, treatments, and diagnoses.

### Participants

Patients were included if they had an index, nonrecurrent CDI hospitalization (International Classification of Diseases, Tenth Revision, Clinical Modification [ICD-10-CM] code A04.72) discharge between July 1, 2019 and December 31, 2022. Prior studies have found that the use of administrative codes (eg, ICD-10-CM) alone is highly sensitive for identifying CDI, particularly among hospitalized patients (> 90%).^18^ The first hospitalization’s discharge date was used to identify the index CDI event. The CDI cohort was further limited to patients 65 years and older to enrich for patients more likely to need skilled nursing care. Patients were excluded if they had an inpatient or outpatient discharge diagnosis for nonrecurrent or recurrent CDI (ICD-10-CM code A04.71 or A04.72) reported within 1 and 90 days prior to the index admission date. Prior hospitalizations were assessed in the 90 days prior to the index visit admission date (beginning April 1, 2019) and subsequent rehospitalizations were assessed in the 90 days following discharge from the index admission (through March 30, 2023).

### Variables

The primary endpoints of this study were admission from or discharge to a SNF. Admission origin was captured using the “point of origin” variable. Discharge location was assessed using the “discharge status” variable, which includes “discharged/transferred to SNF,” “discharged/transferred to a nursing facility certified under Medicaid, but not Medicare,” and “discharged/transferred to a SNF with Medicare certification with a planned acute care hospital readmission.” The “discharge status” variable also includes inpatient mortality and other discharge disposition categories.

Additional patient baseline characteristics included those outlined in Table 1. A patient was considered to have a comorbidity if they had an inpatient or outpatient encounter in the 90 days prior to the index visit or an index CDI visit with a documented ICD-10-CM diagnosis code for the comorbidity (Supplement). Major classes of relevant medications in the 90 days prior to admission or coadministered during the index CDI visit were included (Supplement).

Characteristics related to the CDI diagnosis included principal versus secondary diagnosis, present on admission, and severe CDI (white blood cells ≥ 15 × 10^9^/L or serum creatinine [SCr] > 1.5 mg/dL anytime during the index hospitalization). CDI therapies included metronidazole, oral vancomycin, fidaxomicin, and bezlotoxumab.

Other important CDI-related outcomes were assessed. Current Procedural Terminology (CPT), Healthcare Common Procedure Coding System (HCPCS), and International Classification of Diseases, Tenth Revision, Procedure Coding System (ICD-10-PCS) codes were used to identify complications, including sepsis, surgical intervention related to CDI (eg, subtotal/total colectomy and diverting loop ileostomy), and acute kidney injury (Supplement). CDI-related complications were assessed during the index visit and in the 90 days following the index visit discharge date. Patients with a report of a complication within 90 days prior to the index admission date were excluded from analysis for each complication.

Additional CDI-related outcomes included 90-day all-cause hospital readmissions and CDI-related readmissions (ICD-10-CM codes A04.72 and A04.71). Time to readmission was also captured as a continuous variable. Patients who died during the index visit were excluded from the readmission analysis. Additionally, the principal diagnosis category for all-cause readmission visits was assessed (Supplement). Inpatient mortality was defined using the “discharge status” variable and hospital length of stay (LOS) was assessed using the hospital-submitted “LOS” variable. Lastly, hospital charges included the total charge amount of billed items during the hospital encounter and patient costs included the total cost to treat the patient during the index hospital encounter and during readmissions.

### Statistical methods

Patient baseline characteristics and outcomes were first presented descriptively for the overall population and for those who did and did not have a SNF admission origin and those with and without a SNF discharge. When comparing patients with or without a SNF point of origin, patients with “unknown” origin were excluded from comparative analyses. When comparing patients with or without a SNF discharge disposition, patients with a SNF point of origin, inpatient mortality, or an “unknown” discharge disposition were excluded from comparative analyses. Next, these characteristics were compared between patients with a SNF admission origin or discharge and those without using the χ^2^, Fisher exact, or Wilcoxon rank-sum test as appropriate. Next, we identified predictors of SNF admission and SNF discharge in 2 logistic regression models. Patients with a SNF origin were excluded from the SNF discharge model. To optimize each model, we first limited the characteristics to those present in at least 5% of the overall population. We then assessed multicollinearity between variables using pairwise correlations and the variance inflation factor (VIF). No 2 variables met the threshold of VIF > 10. Next, each baseline characteristic was assessed for its relationship with the outcome using bivariable comparisons; those characteristics that reached *P* < .05 were included in the logistic regression model as covariates. Variables that reached *P* < .05 were considered statistically significant independent predictors in the final models. The SNF admission predictors only included pre-admission or baseline characteristics, whereas the discharge model included factors that occurred during the admission.

## RESULTS

### Population characteristics

A total of 86,646 CDI patients were included in the overall population. Patients were predominantly female, White race, and non-Hispanic ethnicity (Table 1). Three-quarters of CDI diagnoses were secondary diagnoses (74.4%), though CDI was commonly present on admission (77.5%).

Few CDI admissions (5.1%) occurred in patients with a SNF point of origin. CDI patients with a SNF admission origin were older (median age 79 vs 76 years, *P* < .001), more often non-Hispanic (96.0% vs 92.5%, *P* < .001), and located in the Midwest and Northeast regions. SNF CDI admissions were more often emergent (90.7% vs 82.3%, *P* < .001), severe (77.7% vs 71.7%, *P* < .001), and secondary diagnoses (84.0% vs 73.9%, *P* < .001). Comorbidities, most notably dementia, concomitant infections, and prior hospitalizations, were more prevalent among SNF origin patients. Similar trends were observed when comparing CDI patients discharged to SNF compared with non-SNF locations (Supplement).

### Transitions of care

**Fig. 1.**
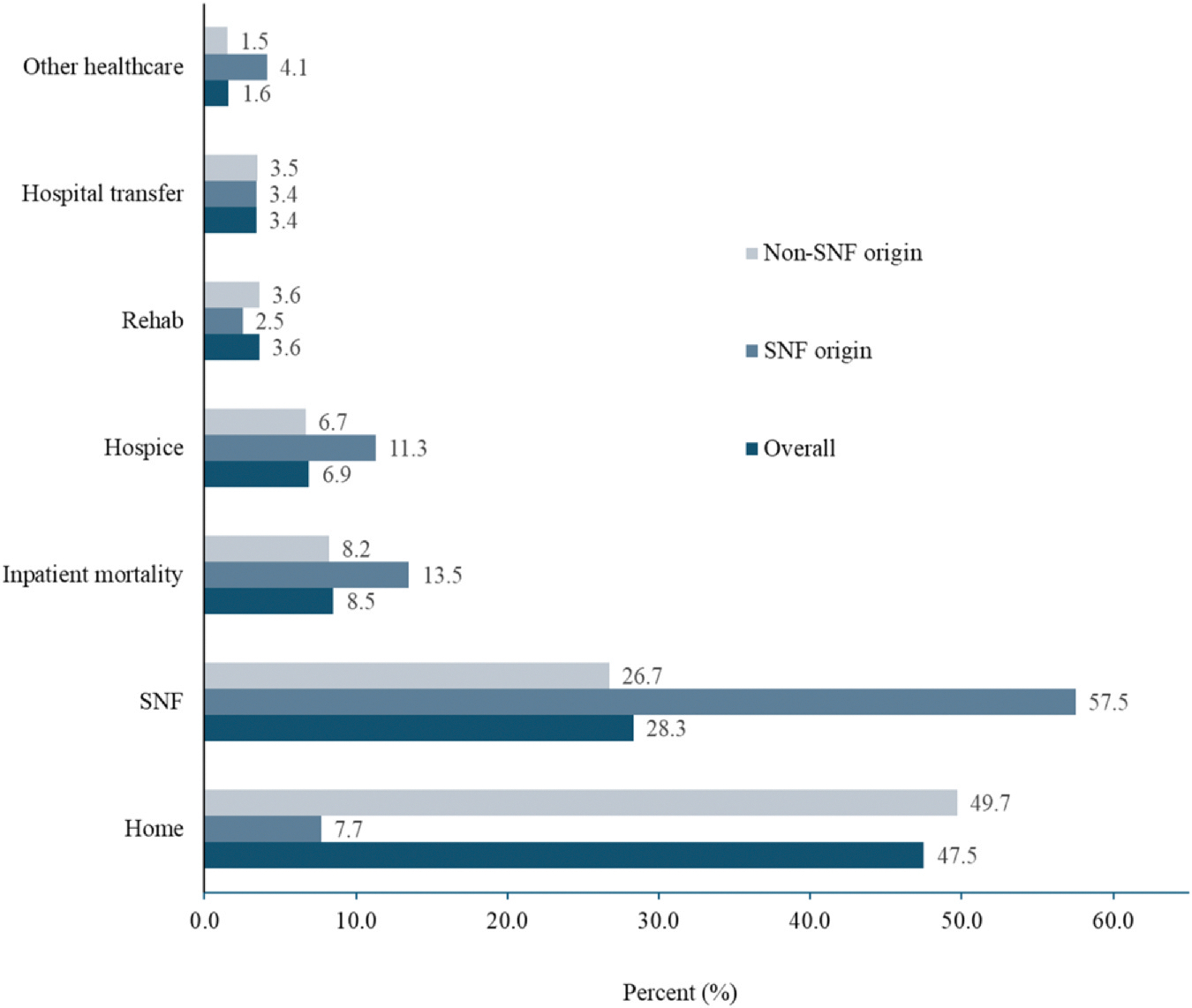
Patient discharge disposition. SNF, skilled nursing facility.

The most common discharge disposition for CDI patients overall was home (47.5%), followed by discharge to a SNF (28.3%) (Fig. 1). Other common dispositions included hospice (6.9%), rehabilitation (3.6%), or another hospital (3.4%). Among patients admitted from a SNF, 57.5% were discharged to a SNF. In contrast, of patients not originally admitted from a SNF, 26.7% were discharged to a SNF. Notably, a small percentage of SNF origin patients are discharged home (7.7%); many of these patients die in the hospital or need additional health care (eg, hospital transfer, rehabilitation, and hospice) if they are not discharged back to a SNF.

Predictors of SNF admission and discharge were similar (Table 2). The strongest predictors of SNF admission included non-Hispanic ethnicity (adjusted odds ratio [aOR] 2.55, 95% CI 1.68–3.89), dementia (aOR 2.56, 95% CI 2.22–2.94), and an emergent CDI admission type (aOR 3.68, 95% CI 2.01–6.72). Dementia was also the strongest predictor of SNF discharge (aOR 1.93, 95% CI 1.75–2.12).

### Patient health and economic outcomes

Patients with a SNF origin were more likely to experience inpatient mortality (13.5% vs 8.2%, *P* < .001), all-cause readmission (31.0% vs 28.0%, *P* < .001), and longer median hospital stays (8 vs 7 days, *P* < .001) (Table 3). Interestingly, patients with a SNF origin were less likely to experience surgical intervention up to 90 days after the index visit (9.3% vs 13.9%, *P* < .001). Patients with a SNF origin also had significantly higher median hospital charges and costs during the index encounter (*P* < .001).

Similar trends were seen in comparisons between CDI patients discharged to SNF and non-SNF locations (Table 3). Patients with a SNF discharge were more likely to experience all-cause hospital readmissions (31.9% vs 26.4%, *P* < .001), longer median time to first all-cause readmission (24 vs 23 days, *P* = .008), longer median time to first CDI readmission (19 vs 16 days, *P* < .001), and longer median hospital stays (10 vs 6 days, *P* < .001). Patients with a SNF discharge also had significantly higher median hospital charges and costs during the index encounter, during all-cause readmissions, and CDI readmissions (*P* < .001 for all comparisons).

The most common readmission principal diagnoses for all-cause readmissions were infectious (22.4%), followed by digestive (20.8%), and circulatory (13.7%) (Supplement). Infectious principal diagnoses were more common among patients admitted from and discharged to a SNF. Excluding CDI readmissions, infectious diagnoses remained the most common principal diagnosis (29.0%), driven by septicemia (24.0% of all non-CDI readmissions).

## DISCUSSION

In this nationally representative and contemporary sample of CDI hospitalizations in older adults, few patients were admitted from a SNF, though over one-quarter of patients were discharged to a SNF. SNF origin and discharge were associated with poorer patient and economic health outcomes, including mortality, longer hospital stays, readmissions, and costs. This study is strengthened by its large, geographically diverse population using robust, real-world electronic health record data.

Our findings highlight the important role of SNFs in CDI transitions of care. First, SNF residents are considered high-risk for CDI due to a higher prevalence of comorbid conditions, health care exposures, and older age compared with community-dwelling older adults. Frailty and poor health status are risk factors for CDI in the elderly. In fact, frailty is more predictive of CDI incidence, mortality, and recurrence compared with chronological age.^19^ One notable finding was the prevalence of dementia among patients originating from and discharged to SNFs. Dementia, particularly more advanced conditions, commonly requires long-term care. In addition, severe infections have been associated with cognitive dysfunction, and infection hospitalization can increase risk of dementia among older adults.^20^

Next, the SNF was a major discharge disposition source. While it is not surprising that most patients originating from a SNF were discharged to a SNF, over one-quarter (28%) of patients admitted from a nonhealth care setting required SNF discharge. Other health care dispositions, including hospital transfer, rehabilitation, and hospice, were common, indicating a higher level of health care need following CDI. Using a national sample of US Veterans with CDI, Reveles et al^12^ found that patients originally admitted from home who developed CDI more often required a nonhome discharge (nursing facility, long-term care, and hospice) compared with non-CDI patients (18% vs 8%, *P* < .001). Nonhome discharge was more common among older patients with severe CDI and chronic comorbidities. Movement of patients between health care facilities can facilitate the dissemination of pathogens, like *C difficile*. CDI discharge from an acute care facility represents a significant risk for importation of *C difficile* to long-term care facilities.^21^ One study found that importation of CDI was 3 times higher in long-term care as compared with acute care,^22^ meaning that there is greater concern for dissemination of cases from acute care to long-term care, rather than from long-term care to acute care.

The need for SNF and other health care services following CDI is complex. First, CDI patients often experience severe or prolonged symptoms. Studies have documented poor quality of life, including physical and mental functioning, during and following a CDI episode.^13,23^ Acute CDI complications can include dehydration, acute kidney injury, gastrointestinal symptoms, and exacerbation of underlying conditions.^14,15^ Patients who experienced fulminant CDI requiring surgery may also require ongoing care. Additional issues that can occur after successful treatment can include fatigue, depression, anxiety, and new-onset gastrointestinal conditions (eg, irritable bowel syndrome). Lurienne et al^14^ reported that more than half of patients continue to experience post-CDI symptoms and 40.9% believed they would never end, highlighting the persistent and debilitating nature of the disease.

Another important finding from this study was the poorer health and economic outcomes among patients presenting from or discharge to a SNF. Over 92% of SNF origin patients were unable to discharge home. Previous studies have found that underlying frailty and cognitive impairment are significant predictors of death after an acute CDI.^23^ In our study, SNF patients were more likely to be older, have severe CDI, dementia, a secondary CDI diagnosis, and an emergent admission. Notably, CDI readmissions were similar between groups (approximately 12%), suggesting that underlying comorbidities or complications from the index CDI hospitalization more commonly contribute to readmission; however, infectious diagnoses were the predominant principal diagnosis for readmissions.

The potential patient, health care, and economic burden associated with SNF residence after CDI discharge is massive. Direct costs include health care services, medications, procedures, and personnel. Indirect costs may be substantial as well due to loss of work or work productivity. Hospital readmissions were common among the overall CDI population, but particularly among those with SNF origin and SNF discharge. Among our CDI cohort, we noted over 30,000 cumulative 90-day readmissions with a median cost ranging from about $10,000 to $15,000 each. CDI and subsequent readmissions can significantly impact hospital quality metrics. In a 2018 publication, Zilberberg et al^16^ reported a $5,000 to $14,000 mean gap in hospital costs and DRG reimbursements between CDI survivors who were readmitted without rCDI and those readmitted with rCDI.

The implications of SNF residence for patients with CDI extend beyond their physical and mental health. For patients originally admitted to the hospital from a SNF, there are ongoing costs associated with reserving their bed during hospitalization, in addition to any hospital copays or deductibles. CDI patients discharged to a SNF may also necessitate enhanced infection control practices, which can strain staff and require additional supplies. Moreover, SNF residents may not have access to preferred antibiotic treatments for CDI, such as fidaxomicin, due to high costs. In our study, oral vancomycin was a significant predictor of SNF discharge, suggesting either a continued need for treatment after discharge or challenges in obtaining fidaxomicin in long-term care settings.

Reimbursement for short-term rehabilitation in SNFs typically comes from Medicare or Medicare Advantage plans. These models offer a fixed daily rate that covers all care, medications, and supplies, making it crucial to keep costs below this threshold. Each of these factors, coupled with the financial implications of potential readmissions, underscores the substantial economic burden of CDI for SNF residents.

Implementing effective multidisciplinary infection control and pharmacologic strategies to prevent CDI and subsequent hospitalizations in the high-risk SNF population is essential. Preventative strategies can include ensuring that SNFs adhere to strict infection control practices, educating SNF staff about CDI transmission risks and protocols, and coordinating with SNFs to implement or strengthen antimicrobial stewardship programs following the CDC’s Core Elements of Antimicrobial Stewardship for Nursing Homes.^24^ Bundled approaches to infection control and prevention,^25^ as well as larger-scale public health interventions have been previously shown to be effective in reducing CDI incidence rates.^26^ Similar to findings from other multidrug-resistant organism studies, limiting high-risk, non-CDI antibiotics in the hospital setting^27^ and implementing enhanced barrier precautions^28^ may help prevent *C difficile* transmission among residents. For patients with suspected CDI, optimizing diagnosis and early initial treatment, especially targeted to recurrence prevention (eg, fidaxomicin, microbiome-targeted therapies), can improve health outcomes. Implementing robust transitions of care and discharge protocols that clearly communicate CDI history and recurrence risk can prompt SNF infection control and monitoring practices and enhance access to optimal follow-up care that includes clinical specialist, nutrition, and hydration support. This approach targets the vulnerabilities of this patient population and addresses the full CDI care continuum.

This study has limitations. Given the retrospective design utilizing electronic medical record data, there is a possibility of misclassifying admission origins, discharge dispositions, and patient and hospital characteristics. CDI was identified through administrative coding, as not all health systems contributed laboratory data for stool test confirmation. The relatively high use of laxatives may suggest that some patients were inaccurately diagnosed. Additionally, while the sample population is nationally representative and geographically diverse, it was limited to older adults, which may affect the generalizability of findings to younger CDI populations outside the PINC AI database. We also lacked specific patient and health care data regarding SNF encounters before or after CDI hospital discharge, preventing us from fully describing the course of CDI therapy in that setting or the exact reasons for health care services after discharge. Lastly, while we identified potential predictors of SNF origin and discharge using multivariable regression, confounding by unmeasured variables cannot be ruled out.

## CONCLUSIONS

Older, hospitalized CDI patients originating from SNFs disproportionately experience poor health outcomes and financial burden. Over one-quarter of CDI patients were discharged to a SNF suggesting a need for higher levels of health care following CDI. SNF residents are an understudied population, particularly on a national level. Further work to describe the CDI incidence and costs associated with skilled nursing care and other health services will be critical to understanding the true economic burden of CDI.

## Supplementary Material

Supplementary Material

## Figures and Tables

**Table 1:** Baseline characteristics, SNF versus non-SNF point of origin

Characteristic	Overall (n = 86,646)	SNF origin (n = 4,416)	Non-SNF origin (n = 81,409)	*P* value

Patient characteristics				
Age (y), median (IQR)	76 (71–83)	79 (73–87)	76 (71–83)	< .001
Female gender, n (%)	50,551 (58.4)	2,611 (59.1)	47,459 (58.3)	.291
White race, n (%)	70,551 (82.8)	3,523 (80.9)	66,337 (82.9)	.001
Hispanic ethnicity, n (%)	5,541 (7.3)	161 (4.0)	5,351 (7.5)	< .001
Medicare payor, n (%)	79,895 (92.2)	4,167 (94.4)	74,978 (92.1)	< .001
Prior hospitalization, n (%)	28,927 (33.4)	2,134 (48.3)	26,521 (32.6)	< .001
Prior comorbidities, n (%)				
Myocardial infarction	14,794 (17.1)	836 (18.9)	13,823 (17.0)	.001
Congestive heart failure	32,358 (37.3)	2,054 (46.5)	30,018 (36.9)	< .001
Peripheral vascular disease	16,134 (18.6)	845 (19.1)	15,128 (18.6)	.358
Cerebrovascular disease	10,988 (12.7)	800 (18.1)	10,086 (12.4)	< .001
Dementia	16,446 (19.0)	1,770 (40.1)	14,537 (17.9)	< .001
Chronic pulmonary disease	27,254 (31.5)	1,461 (33.1)	25,519 (31.3)	.015
Rheumatic disease	5,124 (5.9)	231 (5.2)	4,862 (6.0)	.042
Peptic ulcer disease	3,807 (4.4)	221 (5.0)	3,536 (4.3)	.037
Mild liver disease	8,707 (10.0)	319 (7.2)	8,304 (10.2)	< .001
Moderate/severe liver disease	2,361 (2.7)	84 (1.9)	2,255 (2.8)	.001
Diabetes without complications	24,875 (28.7)	1,521 (34.4)	23,154 (28.4)	< .001
Diabetes with complications	25,674 (29.6)	1,571 (35.6)	23,876 (29.3)	< .001
Paraplegia/hemiplegia	2,777 (3.2)	221 (5.0)	2,532 (3.1)	< .001
Renal disease	38,408 (44.3)	2,246 (50.9)	35,806 (44.0)	< .001
Cancer	15,734 (18.2)	522 (11.8)	15,068 (18.5)	< .001
Metastatic cancer	5,875 (6.8)	180 (4.1)	5,651 (6.9)	< .001
AIDS/HIV	139 (0.2)	7 (0.2)	130 (0.2)	.985
Hypertension	75,205 (86.8)	3,957 (89.6)	70,541 (86.7)	< .001
Dyslipidemia	54,452 (62.8)	2,878 (65.2)	51,084 (62.8)	.001
Obesity	17,793 (20.5)	1,013 (22.9)	16,628 (20.4)	< .001
GERD	29,104 (33.6)	1,625 (36.8)	27,200 (33.4)	< .001
IBD	3,521 (4.1)	133 (3.0)	3,361 (4.1)	< .001
IBS	2,085 (2.4)	87 (2.0)	1,972 (2.4)	.056
CCI Score, median (IQR)	4(2–6)	4(2–6)	4 (2–6)	< .001
Common concomitant infections, n (%)				
Pneumonia	15,102 (17.4)	1,000 (22.6)	13,942 (17.1)	< .001
Urinary tract infection	24,803 (28.6)	1,837 (41.6)	22,742 (27.9)	< .001
Skin infection	5,268 (6.1)	277 (6.3)	4,945 (6.1)	.593
Prior medication classes, n (%)				
Antimicrobials (non-CDI)	35,693 (41.2)	2,344 (53.1)	33,021 (45.6)	< .001
Gastric acid suppressants	25,830 (29.8)	1,688 (38.2)	23,905 (29.4)	< .001
Antineoplastic/immunomodulators	3,798 (4.4)	155 (3.5)	3,606 (4.4)	.003
Immunosuppressants	844 (1.0)	35 (0.8)	806 (1.0)	.180
Laxatives/stool softeners	16,664 (19.2)	1,354 (30.7)	15,166 (18.6)	< .001
Antidiarrheal	3,127 (3.6)	182 (4.1)	2,920 (3.6)	< .001
Probiotics	3,547 (4.1)	286 (6.5)	3,201 (3.9)	< .001
Opioid pain medications	27,295 (31.5)	1,599 (36.2)	25,428 (31.2)	< .001
GI IBS anti-/promotility	7,276 (8.4)	412 (9.3)	6,813 (8.4)	.027
Concomitant medication classes, n (%)				
Antimicrobials (non-CDI)	69,376 (80.1)	3,846 (87.1)	64,856 (79.7)	< .001
Gastric acid suppressants	60,105 (69.4)	3,062 (69.3)	56,513 (69.4)	.911
Antineoplastic/immunomodulators	5,100 (5.9)	228 (5.2)	4,838 (5.9)	.030
Immunosuppressants	1,774 (2.0)	45 (1.0)	1,722 (2.1)	< .001
Laxatives/stool softeners	24,147 (27.9)	1,391 (31.5)	22,555 (27.7)	< .001
Antidiarrheal	6,649 (7.7)	232 (5.3)	6,359 (7.8)	< .001
Probiotics	17,683 (20.4)	935 (21.2)	16,524 (20.3)	.161
Opioid pain medications	50,389 (58.2)	2,453 (55.5)	47,487 (58.3)	< .001
GI IBS anti-/promotility	13,898 (16.0)	558 (12.6)	13,222 (16.2)	< .001
Hospitalization and CDI characteristics				
Urban setting, n (%)	75,191 (86.8)	3,752 (85.0)	70,846 (87.0)	< .001
Teaching hospital, n (%)	40,825 (47.1)	2,379 ( 53.9)	38,278 (47.0)	< .001
Region, n (%) South	38,371 (44.3)	1,494 (33.8)	36,527 (44.9)	< .001
Midwest	20,428 (23.6)	1,367 (31.0)	19,005 (23.3)	
Northeast	14,058 (16.2)	1,163 (26.3)	12,873 (15.8)	
West	13,789 (15.9)	392 (8.9)	13,004 (16.0)	
Provider specialty, n (%)				< .001
Family/internal medicine	33,045 (38.1)	1,654 (37.5)	30,962 (38.0)	
Hospitalist	27,172 (31.4)	1,541 (34.9)	25,384 (31.2)	
Critical care	1,839 (2.1)	158 (3.6)	1,661 (2.0)	
Emergency medicine	1,284 (1.5)	95 (2.2)	1,161 (1.4)	
Infectious disease	145 (0.2)	8 (0.2)	137 (0.2)	
Gastroenterology	124 (0.1)	4 (0.1)	119 (0.1)	
Other specialist	8,895 (10.3)	305 (6.9)	8,532 (10.5)	
Unknown	14,142 (16.3)	651 (14.7)	13,453 (16.5)	
CDI diagnosis type, n (%)				< .001
Principal	22,162 (25.6)	705 (16.0)	21,252 (26.1)	
Secondary	64,484 (74.4)	3,711 (84.0)	60,157 (73.9)	
Non-CDI principal diagnoses, n (%)				
Infectious/parasitic	26,932 (41.8)	1,955 (52.7)	24,718 (41.1)	
Circulatory system	7,392 (11.5)	286 (7.7)	7,035 (11.7)	
Injury/poisoning	6,324 (9.8)	386 (10.4)	5,884 (9.8)	
Digestive system	5,680 (8.8)	224 (6.0)	5,416 (9.0)	
Genitourinary system	4,017 (6.2)	205 (5.5)	3,778 (6.3)	
Respiratory system	3,123 (4.8)	196 (5.3)	2,892 (4.8)	
CDI present on admission, n (%)	66,978 (77.5)	3,442 (78.2)	62,894 (77.5)	.219
Severe CDI, n (%)	13,312 (72.1)	877 (77.7)	12,410 (71.7)	< .001
CDI therapies, n (%)				
Metronidazole	35,690 (41.2)	1,852 (41.9)	33,492 (41.1)	.294
Oral vancomycin	75,127 (86.7)	3,793 (85.9)	70,623 (86.8)	.102
Fidaxomicin	5,799 (6.7)	301 (6.8)	5,445 (6.7)	.741
Bezlotoxumab	31 (0.04)	0 (0)	31 (0.04)	.407
Admission type, n (%)				< .001
Emergency	71,373 (82.7)	3,987 (90.7)	66,705 (82.3)	
Urgent	11,144 (12.9)	330 (7.5)	10,707 (13.2)	
Elective	3,180 (3.7)	39 (0.9)	3,127 (3.9)	
Trauma center	604 (0.7)	39 (0.9)	554 (0.7)	

*AIDS*, acquired immunodeficiency syndrome; *CCI*, Charlson Comorbidity Index; *GERD*, gastroesophageal reflux disease; *GI*, gastrointestinal; *HIV*, human immunodeficiency virus; *IBD*, irritable bowel disease; *IBS*, irritable bowel syndrome; *SNF*, skilled nursing facility.

**Table 2:** Predictors of SNF admission origin and discharge

Characteristic	SNF origin aOR (95% CI)	*P* value	SNF discharge aOR (95% CI)	*P* value

Patient characteristics				
Age per unit change	1.03 (1.02–1.04)	< .001	1.03 (1.03–1.04)	< .001
White race	1.05 (0.87–1.26)	.612	0.95 (0.85–1.06)	.367
Non-Hispanic ethnicity	2.55 (1.68–3.89)	< .001	1.37 (1.14–1.63)	< .001
Medicare payor	1.30 (0.99–1.71)	.059	1.28 (1.10–1.48)	< .001
Prior hospitalization	1.07 (0.84–1.36)	.590	1.08 (1.00–1.18)	.061
Comorbidities				
Myocardial infarction	1.03 (0.87–1.21)	.769	0.93 (0.84–1.03)	.163
Congestive heart failure	1.11 (0.96–1.28)	.166	1.09 (1.00–1.19)	.043
Cerebrovascular disease	1.15 (0.97–1.37)	.116	1.24 (1.11–1.39)	< .001
Dementia	2.56 (2.22–2.94)	< .001	1.93 (1.75–2.12)	< .001
Chronic pulmonary disease	1.02 (0.89–1.17)	.779	1.10 (1.01–1.20)	.020
Rheumatic disease	0.96 (0.73–1.26)	.762	0.92 (0.78–1.08)	.285
Liver disease	0.71 (0.54–0.91)	.008	0.95 (0.84–1.00)	.465
Diabetes	1.31 (1.14–1.52)	< .001	1.07 (0.99–1.17)	.102
Renal disease	0.97 (0.84–1.12)	.639	1.00 (0.92–1.10)	.999
Cancer	0.67 (0.55–0.82)	< .001	0.72 (0.65–0.80)	< .001
Hypertension	0.93 (0.75–1.16)	.530	0.98 (0.87–1.11)	.783
Dyslipidemia	0.96 (0.83–1.11)	.550	0.99 (0.90–1.07)	.727
Obesity	1.15 (0.98–1.36)	.081	1.23 (1.12–1.35)	< .001
Medication class*				
Non-CDI antimicrobials	1.24 (1.01–1.53)	.037	1.29 (1.15–1.44)	< .001
Gastric acid suppressants	1.03 (0.85–1.25)	.741	1.08 (0.99–1.18)	.067
Antineoplastic/immunomodulators	1.14 (0.81–1.60)	.465	–	–
Immunosuppressant	–	–	0.63 (0.47–0.87)	.004
Laxatives/stool softeners	1.41 (1.17–1.70)	< .001	1.50 (1.38–1.64)	< .001
Opioid pain medications	–	–	1.06 (0.97–1.15)	.178
Probiotics	1.37 (1.04–1.80)	.025	1.01 (0.92–1.10)	.833
Oral vancomycin	–	–	1.40 (1.19–1.65)	< .001
Hospitalization and CDI characteristics				
Rural versus urban setting	1.09 (0.90–1.32)	.377	0.83 (0.74–0.93)	< .001
Teaching hospital	1.31 (1.14–1.51)	< .001	1.00 (0.92–1.09)	.937
Region				
South	1.00 (reference)		1.00 (reference)	
Northeast	2.20 (1.84–2.62)	< .001	1.34 (0.19–1.50)	< .001
Midwest	1.64 (1.40–1.92)	< .001	1.18 (1.08–1.30)	< .001
West	0.96 (0.50–1.85)	.903	0.82 (0.59–1.13)	.223
Admission type				
Elective	1.00 (reference)		1.00 (reference)	
Urgent	0.96 (0.48–1.92)	.905	1.14 (0.91–1.43)	.247
Emergency	3.68 (2.01–6.72)	.002	1.26 (1.04–1.54)	.075
Trauma	3.44 (1.32–8.94)	.902	1.56 (0.96–2.56)	.075
Secondary CDI diagnosis	1.52 (1.28–1.80)	< .001	1.44 (1.29–1.59)	< .001
Severe CDI	1.17 (0.99–1.37)	.058	1.11 (1.01–1.22)	.027
CDI present on admission	–	–	0.82 (0.74–0.90)	< .001
Surgical intervention	–	–	0.87 (0.77–0.96)	.007
Sepsis	–	–	1.08 (0.99–1.18)	.083
Acute kidney injury	–	–	1.12 (1.03–1.22)	.007

*aOR, adjusted odds ratio*; *CDI, Clostridioides difficile* infection; *SNF*, skilled nursing facility.

* Medication class represents prior medications for SNF origin analysis and concomitant medications for SNF discharge analysis.

**Table 3:** Patient health and economic outcomes, SNF versus non-SNF point of origin and discharge

Outcome	SNF origin (n = 4,416)	Non-SNF origin (n = 81,409)	*P* value	SNF discharge (n = 21,968)	Non-SNF discharge (n = 53,358)	*P* value
Complications, n (%)						
Acute kidney injury	2,487 (56.3)	41,430 (50.9)	<.001	11,983 (54.5)	24,868 (46.6)	<.001
Sepsis	2,638 (59.7)	36,336 (44.6)	<.001	10,603 (48.3)	20,782 (38.9)	<.001
Surgical intervention	412 (9.3)	11,347 (13.9)	<.001	2,755 (12.5)	7,955 (14.9)	<.001
All-cause readmission, n (%)	1,185 (31.0)	20,891 (28.0)	<.001	7,017 (31.9)	14,063 (26.4)	<.001
Time to first all-cause readmission (d), median (IQR)	23 (10–44)	23 (11–45)	.678	24 (11–45)	23 (10–44)	.008
Any CDI readmission, n (%)	458 (12.0)	8,889 (11.9)	.875	2,679 (12.2)	6,299 (11.8)	.134
Time to first CDI readmission (d), median (IQR)	17.5 (7–31)	17 (7–31)	.523	19 (8–33)	16 (6–30)	<.001
Inpatient mortality, n (%)	594 (13.5)	6,704 (8.2)	<.001	–	–	–
Hospital LOS, median (IQR)	8 (5–14)	7 (4–13)	<.001	10 (6–16)	6 (4–11)	<.001
Hospital charges, median (IQR)	64,745 (36,110–125,354)	58,947 (31,800–122,581)	<.001	75,945 (41,787–145,849)	48,936 (27,638–97,677)	<.001
Hospital costs, median (IQR)	18,610 (10,193–34,078)	15,270 (8,162–31,148)	<.001	20,546 (11,597–38,281)	12,329 (6,950–24,839)	<.001
Readmission economic outcomes*	SNF origin (n = 2,993)	Non-SNF origin (n = 34,278)	*P* value	SNF discharge (n = 7,139)	Non-SNF discharge (n = 18,839)	*P* value
All-cause readmission charges, median (IQR)	55,367 (31,040–112,445)	53,064 (29,306–107,472)	.092	55,380 (32,570–97,057)	41,042 (24,461–74,083)	<.001
All-cause readmission costs, median (IQR)	15,101 (8,436–28,463)	13,582 (7,438–27,411)	<.001	14,784 (8,572–25,597)	10,438 (6,236–18,534)	<.001
All CDI readmission charges, median (IQR)	62,236 (33,498–133,959)	65,060 (33,276–148,140)	.080	56,405 (32,899–100,806)	40,475 (24,036–74,101)	<.001
All CDI readmission costs, median (IQR)	17,393 (9,259–34,632)	16,939 (8,540–37,462)	.434	15,449 (8,962–27,395)	10,653 (636–18,956)	<.001

*CDI, Clostridioides difficile* infection; *LOS*, length of stay; *SNF*, skilled nursing facility.

* Cumulative readmissions for all unique patients over the 90-day follow-up period.

## APPENDIX A. SUPPLEMENTARY DATA

Supplementary data related to this article can be found at doi:10.1016/j.ajic.2025.10.012.
