# Supplementary material for: Role of the skilled nursing facility in Clostridioides difficile infection transitions of care: A retrospective cohort study of US hospitals
Source: Am J Infect Control. Author manuscript; Available in PMC 2026 Jun 17. (PMC13274802; doi:10.1016/j.ajic.2025.10.012)

Supplement

Supplement 1. Summary of study dependent variables

Dependent variable	Definition
All-cause mortality	Death as indicated in the VA Vital Status File
Frailty-associated diagnoses	Indicated by ICD-9 or ICD-10 code below
Coagulopathy	286.0-286.9 (ICD-9) or D65-D69 (ICD-10)
Involuntary weight loss	783.21 (ICD-9) or R63.4 (ICD-10)
Fluid & electrolyte imbalance	276.9 (ICD-9) or E87 (ICD-10)
Anemia	280.0-285.9 (ICD-9) or D60-D64 (ICD-10)
Falls	V15.88 (ICD-9) or Z91.81 (ICD-10)
Fracture	800.0-829.9 (ICD-9) or S02, S22, S32, M48 (ICD-10)
VA Frailty Index	As described by Cheng et al.[20]

Supplement 2. Time-to-death for CDI patients compared to matched controls

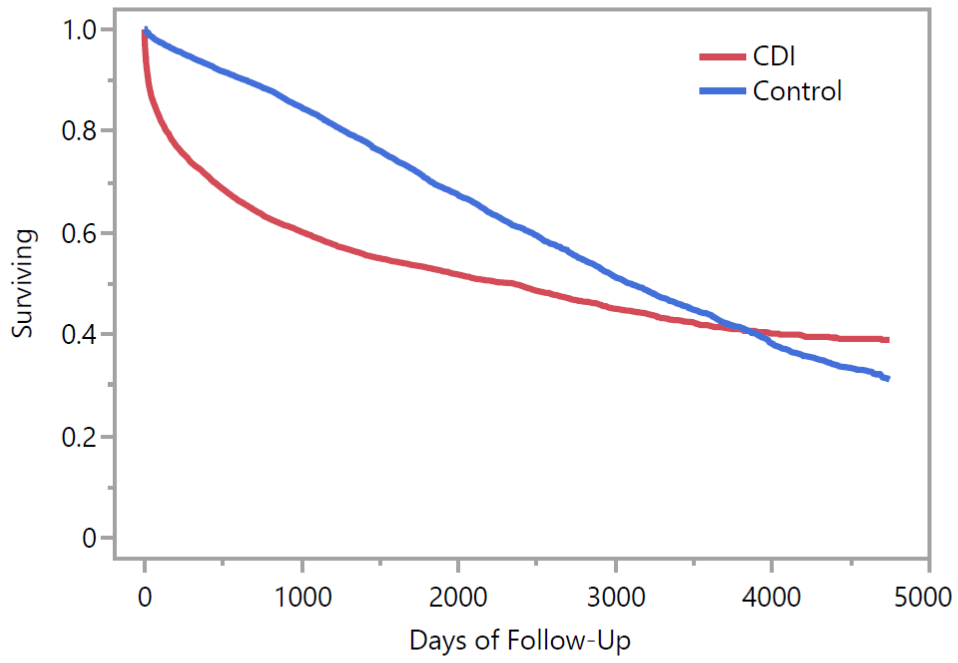

Supplement 3. Time-to-death for CDI patients matched by frailty category

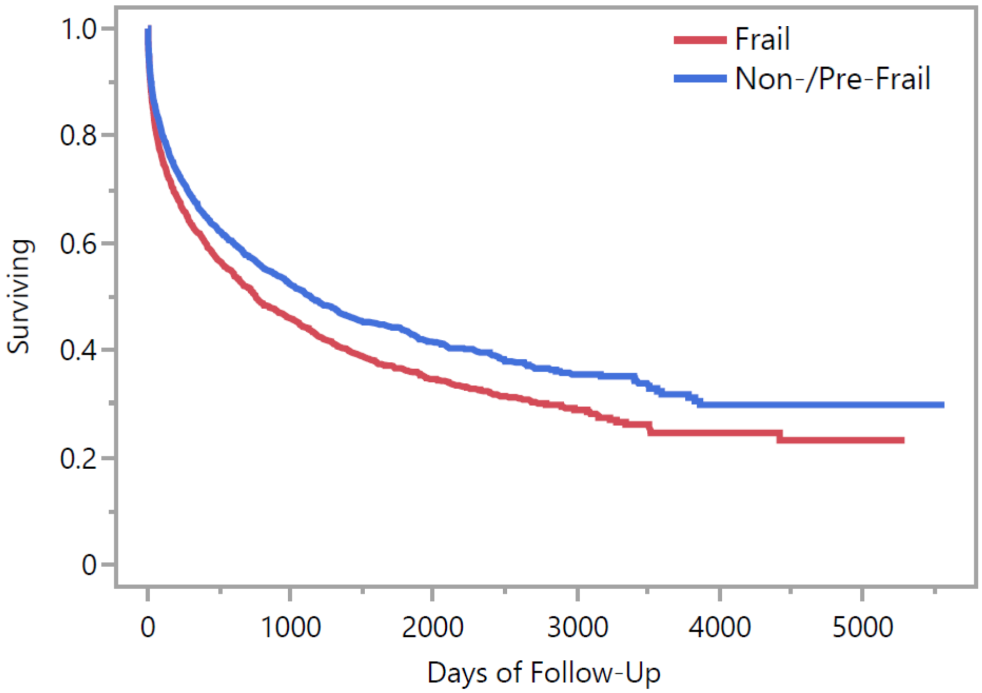

Supplement: Supplementary Material [file NIHMS2178988-supplement-Supplementary_Material.pdf]
